# Implementation of a couple-based HIV prevention program: a cluster randomized trial comparing manual versus Web-based approaches

**DOI:** 10.1186/s13012-014-0116-x

**Published:** 2014-09-11

**Authors:** Susan S Witte, Elwin Wu, Nabila El-Bassel, Timothy Hunt, Louisa Gilbert, Katie Potocnik Medina, Mingway Chang, Ryan Kelsey, Jessica Rowe, Robert Remien

**Affiliations:** Social Intervention Group, Columbia University School of Social Work, 1255 Amsterdam Ave, New York, NY 10027 USA; Center for New Media Teaching and Learning (CCNMTL), Columbia University, 535 W. 114th St, Suite 505, New York, NY 10027 USA; HIV Center for Clinical and Behavioral Studies, New York State Psychiatric Institute and Columbia University, 1051 Riverside Drive, New York, NY 10032 USA

**Keywords:** Couple-based, Implementation, HIV prevention, Multimedia, Web-based

## Abstract

**Background:**

Despite great need, the number of HIV prevention implementation studies remains limited. The challenge for researchers, in this time of limited HIV services agency resources, is to conceptualize and test how to disseminate efficacious, practical, and sustainable prevention programs more rapidly, and to understand how to do so in the absence of additional agency resources. We tested whether training and technical assistance (TA) in a couple-based HIV prevention program using a Web-based modality would yield greater program adoption of the program compared to training and TA in the same program in a manual-based modality among facilitators who delivered the interventions at 80 agencies in New York State.

**Methods:**

This study used a cluster randomized controlled design. Participants were HIV services agencies (N = 80) and up to 6 staff members at each agency (N = 253). Agencies were recruited, matched on key variables, and randomly assigned to two conditions. Staff members participated in a four-day, face-to-face training session, followed by TA calls at two and four months, and follow-up assessments at 6, 12, and 18 months post- training and TA. The primary outcomes examined number of couples with whom staff implemented the program, mean number of sessions implemented, whether staff implemented at least one session or whether staff implemented a complete intervention (all six sessions) of the program. Outcomes were measured at both the agency and participant level.

**Results:**

Over 18 months following training and TA, at least one participant from 13 (33%) Web-based assigned agencies and 19 (48%) traditional agencies reported program use. Longitudinal multilevel analysis found no differences between groups on any outcomes at the agency or participant level with one exception: Web-based agencies implemented the program with 35% fewer couples compared with staff at manual-based agencies (IRR 0.35, CI, 0.13-0.94).

**Conclusion:**

Greater implementation of a Web-based program may require more resources and staff exposure, especially when paired with a couple-based modality. Manual-based and traditional programs may hold some advantage or ease for implementation, particularly at a time of low economic resources.

**Trial registration:**

ClinicalTrials.gov identifier: NCT01863537

## Background

Despite the need for implementation science in HIV prevention in the United States, the number of implementation studies remains limited [1–4]. This may be due to the fact that while there is urgency for HIV scientists to identify, demonstrate and promote robust implementation models, the prevention field is experiencing a shift in scientific priorities [5,6]. Shifts include movement from behavioral towards biomedical prevention approaches, promotion of behavioral couple-based models to overcome barriers in traditional, individually-based models, a recognition that efficacious multisession programs need to be briefer for sustainability in service settings [7] and that programming should target individuals living with HIV and their intimate partners [6]. Historically, the U.S. is also slowly rebounding from an economic recession. This context, in light of the traditional barriers to implementation efforts in real world HIV services agencies, may be holding back more rapid gains in HIV prevention implementation science in the U.S.

The challenge for researchers, in this time of limited HIV services agency resources, is to conceptualize and test how to more rapidly disseminate cost-effective, efficacious, practical, and sustainable prevention programs- including behavioral approaches [8]. We cannot abandon behavioral approaches because even as biomedical innovations for HIV prevention show great promise in efficacy trials, they likely cannot be fulfilled without behavioral interventions to support their adoption and dissemination [9]. Barriers evident in individual level prevention approaches have led to increased testing of couple-based strategies which are often found to be more efficacious in promoting HIV counseling and testing [10,11] and supporting medication adherence [12], but which may require more complex implementation strategies [13,14]. Given the promise of couple-focused approaches (*e.g.*, balancing attention to the dyad, positive reinforcement for relationship-based behaviors, conflict management) [15], scientists have called for improved efforts at examining their dissemination [13]. Yet, couple-based services present a unique set of implementation challenges [13,14]. How can we best balance complexity with ease of access or more rapid dissemination?

Dissemination of evidence-based HIV prevention in the U.S. has taken place largely through the Centers for Disease Control and Prevention’s (CDC) Diffusion of Evidence-Based Interventions (DEBI) program in which agencies receive funding and technical assistance (TA) to deliver ‘packaged’ programs that had been selected as ‘best evidence’ [16,17]. This national approach is based on best evidence from Kelly *et al.* [18,19] and offers paper-based manuals, training workshops, and follow-up TA. TA may be formally or informally provided, and includes advice, assistance and training pertaining to the implementation and maintenance of the program. Since then there are a number of studies which examine the conduct and monitoring processes of agencies funded to implement CDC DEBI packages [20–24], but few have examined actual implementation of any DEBI program [1,25] and none have tested alternative, scalable approaches to disseminating these packaged interventions. At the same time, computer-assisted and Internet-based prevention programs have demonstrated better short- and long-term efficacy for a range of outcomes [26–28] compared to manually based approaches, including HIV prevention risk reduction [29,30]. Advantages of Web-based technologies are that they may expedite the use of effective interventions in real-world settings [30,31] by offering the potential for time efficiency, cost-effectiveness, scalability, and enhanced learning via Internet-based approaches [32–35]. Scientists are calling for experiments testing computer and Internet-based dissemination formats on implementation [7,19,30,36–41].

This clinical trial tested the adoption of an HIV prevention intervention model to clinic-based health services in neighborhoods throughout New York State. The goal of the study was to examine whether using a Web-based approach for implementation of a couple-focused HIV prevention program, Connect, yields greater implementation than a traditional, manual-based approach used by facilitators at HIV service agencies. We tested implementation of couple-based intervention modalities (manual versus Web-based) into real world settings. The study investigated how the Internet may be used to support the delivery of a behavioral ‘packaged’ manual-based intervention and ‘packaged’ computer based intervention in agency settings [30]. Although both manual-based and Web-based approaches require a facilitator for implementation, we hypothesized that the Web-based approach might ease the implementation process, leading to increased use. Compared to manual-based approaches, Web-based program activities may offer greater ease of access to program and re-training materials, increase the number of facilitators who could train on and use the program following an initial training, and increase rehearsal and self-efficacy for implementing activities among those facilitators who use the program [42]. We had four sets of hypotheses regarding whether more agencies (or participants) assigned to the Web-based program would implement the program (*e.g.*, delivered sessions). We hypothesized that more agencies (or participants) assigned to implement the Web-based program would report H_1_, delivering Connect with more couples; H_2_, delivering more Connect sessions; H_3_, delivering at least one Connect session; and H_4_, delivering at least one complete Connect intervention (all six sessions) over the 18 months of follow up, compared to agencies (and staff) assigned to the manual-based approach. In addition we observed post-training use of TA and barriers to implementation.

## Methods

### Study design

**Figure 1:**
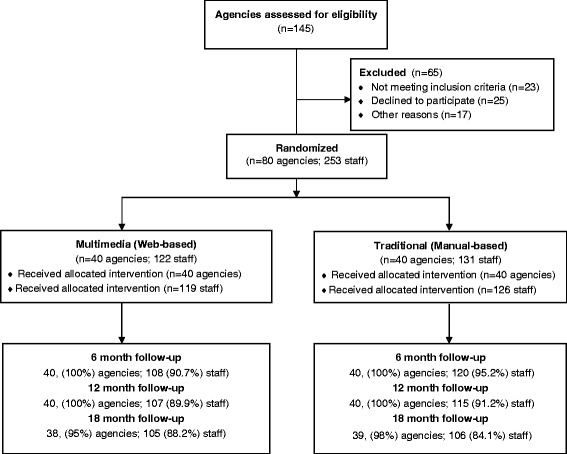
**Study design flowchart of intervention testing Web-based versus manual-based implementation of a couple-focused HIV prevention intervention.**

This study was a longitudinal matched pairs cluster randomized trial conducted between 2007 and 2012 [43,44]. We recruited agencies providing HIV services in New York State. Eligible agencies had ‘not-for-profit’ status in the U.S., provided HIV prevention services to heterosexual men and women, and agreed to send at least one participating staff member to receive training on one of two versions of the Connect program (traditional or Multimedia). We screened 145 agencies identified from a list of state-funded community service providers (CSPs) and multi-service agencies (MSAs) as well as non-state funded agencies identified through five Web sites of HIV services coalitions. The final sample was made up of 80 agencies (see Figure 1).

HIV services agencies are highly variable in terms of their size, scope of service, and available financial resources. Given this variability, and the relatively small sample size, we decided that a matched pair approach would best guard against imbalance across arms after random assignment. We identified two factors that the research team, in consultation with our community advisory board (CAB), believed might most strongly influence the primary outcomes: number of full and part time paid staff providing HIV prevention services in the prior year; and number of clients receiving multi-session HIV prevention services in the prior year. To create matches for pairing [43,44], the project statistician rank ordered and categorized these two factors (in ascending order) from agency level screening assessments (completed by agency Directors) to create a two-way table with an even-number frequency in each cell. The statistician then generated a random number for each case: standard normal (Gaussian) random variates. He then sorted by random numbers in each cell and assigned every two cases as one pair in order. The first case was assigned to the Multimedia condition; the second to the Traditional condition as described below.

### Intervention

#### Manual-based intervention package

Participants from agencies randomized to the manual-based intervention were invited to a four-day, face-to-face structured orientation and training on the original, manualized Connect program. In addition, we offered planned, investigative-team-initiated telephone consultations to provide TA at two and four months following the workshop. The traditional Connect program and facilitator training curriculum is one of the CDC DEBIs (see http://www.effectiveinterventions.org/en/HighImpactPrevention/Interventions/Connect.aspx) [43,44] and is provided in six weekly, two-hour sessions by a single facilitator to a couple using a standard sequence of guided activities. Connect is theoretically and empirically derived, grounded in participatory research [45], with demonstrated efficacy to reduce risk among HIV-negative, HIV-positive or serodiscordant heterosexual couples [46–50]. Connect focuses on the relationship as the target of change; its core elements include redefining risk from individual protection to preserving the relationship; examining issues of fidelity, gender differences, power, and decision making; and using modeling, role-play, and feedback to promote mastery of skills that enhance risk reduction behavior, including couple communication, negotiation, problem solving, and social-support enhancement. Connect has been adapted and replicated with efficacy in a number of trials domestically and globally with youth, men who have sex with men (MSM) couples, drug-affected, and HIV serodiscordant African American couples [51–55].

#### Web-based intervention package

**Figure 2:**
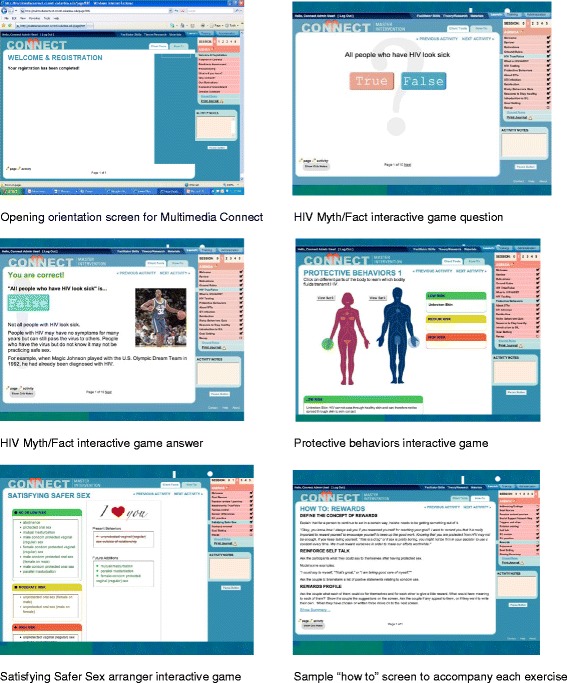
**Screenshots of Multimedia Connect program.**

Participants from agencies randomized to the Web-based intervention were invited to a four-day, face-to-face structured orientation and training on the ‘Multimedia Connect’ program (translated from the original into a Web-based format). In addition, we offered planned, investigative-team–initiated telephone consultations to provide TA at two and four months following the workshop. The Multimedia Connect program and facilitator training is a translation of the original, which features the same core elements [19], but replaces hard copy materials with a Web-based interface of translated interactive tools and video enhancements a facilitator uses as a ‘road map’ to lead participants through activities (see Figure 2). This version also dynamically monitors session progress (*e.g.*, time spent on which activity, when, and by whom). The translation from manual-based intervention to multimedia version was based on design research methodology [56] and scaffolded learning theory [57], and is described in detail elsewhere [58,59]. The goals of this version were to simplify facilitation so that staff with a wider range of educational and experiential levels, from peer volunteers to trained professionals, could use the program; to increase the couple’s engagement with the materials; and to make it easy to rapidly deploy and disseminate to agencies via the Internet.

### Conceptual framework

Study implementation was guided by social cognitive and scaffolded learning theories. Social cognitive theory [18,60] suggests implementation of a new program occurs when there is exposure to and motivation among agency staff to acquire the skills and resources to enact the program; the skills needed to implement the program are acquired through instruction, modeling, and rehearsal; and feedback and reinforcement are provided during the early phases of implementation. Reinforcement, in turn, is enhanced through scaffolded learning, which allows learners to revisit content as needed until they acquire new skills [57]. Building the multimedia facilitator training into the same Web-based environment as the program and offering numerous retraining resources for facilitators present many opportunities for scaffolded learning.

We provided scheduled program trainings once or twice per month on a rolling basis for groups of up to five agencies (15 – 20 staff participants) from the same condition (manual-based or Web-based). The study was longitudinal with repeated measurement of outcome variables at 6, 12, and 18 months post-training and TA.

### Technical assistance

TA was offered in two ways. As part of each condition, TA was initiated by the research team to each agency at two and four months post-program training. During these hour-long conference calls, participants were asked a series of questions regarding their efforts to implement the program and asked questions regarding any barriers or issues faced. The research team provided support and problem-solving strategies to each issue or question raised. In addition to this TA as part of the process, we also encouraged all participants to call or email at any time throughout the trial if they had any need for support related to implementation of the program. These ‘proactive’ efforts on the part of participants were all recorded as they could also possibly influence the implementation outcomes.

As recommended in the literature, we employed a community advisory board (CAB) representing administrators, clinicians, and clients from HIV prevention organizations [61] to assure study protocols were relevant and appropriate for agency-based settings [62].

### Assessment

Prior to being trained in the program’s implementation, individual staff participants completed informed consent and an online baseline assessment of demographic and organizational characteristics, descriptive variables, outcome variables and barriers to implementation. The primary study outcomes were measures of implementation of the Connect program (*e.g.*, delivered sessions), operationalized as follows: the total number of couples with whom staff implemented Connect at each agency; the mean number of Connect sessions implemented by staff at each agency whether staff implemented at least one session of Connect; and whether staff implemented a complete cycle of Connect in the prior six months. Early implementation of this type (also known as ‘adoption’) offers a window into early use and barriers to use of new programs [63,64]. In the original research design, outcomes were at the agency-level [65]. Barriers to implementation were assessed at the 18-month follow-up by asking participants to ‘check each item that may be a barrier to implementing CONNECT at your agency’, asking for endorsement of as many as apply for them at their agency. Examples of potential barriers to endorse included ‘lack of funds’, ‘inadequate staff’, or ‘inadequate training in intervention’.

Figure 1 presents the study design and flow.

### Incentives

Each participating agency received a laptop computer and $250 stipend to offset costs incurred due to participation in training and interviews. Each participating staff received $25, $30, $35, and $40 in compensation for completion of baseline, 6, 12, and 18 month assessments, respectively. An additional incentive was that the training qualified the staff member for Certified Alcohol and Substance Abuse Counselor continuing education credit.

### Sample size calculations and data analysis

Power analyses--assuming alpha = 0.05, two-tailed testing, minimum ‘small-to-medium’ [66] effect size f^2^ = 0.10, and variance inflation factors (VIFs) ranging from 1.2 to 1.5 to account for the design effect resulting from use of matched pairs during randomization--suggested that 80% power would be achieved with 80 agencies as the unit of analysis.

Organizational characteristics and stratification factors were analyzed to check for balance and proper application of the random assignment process. Demographic data were analyzed for differences between participants from the two conditions.

We relied on an intent-to-treat approach and we used generalized linear models with random effects for repeated measures to estimate differences attributed to assignment (Web-based = 1, Manual-based = 0) for four primary outcomes at the 6-, 12-, and 18-month follow up: the total number of couples with whom staff implemented Connect at each agency (negative binomial regression); the mean number of Connect sessions implemented by staff at each agency (linear regression); whether staff implemented at least one session of Connect (logistic regression); and whether staff implemented a complete cycle of Connect (logistic regression). Originally, the agency was the unit of analysis [65].

To be consistent with the agency level analyses, we used generalized linear models with random effects for repeated measures and agency to account for the clusters of individuals within agencies. Outcomes were operationalized as above, but measured individually: the total number of couples with whom participant implemented Connect at his/her agency; sum of the number of Connect sessions implemented by participant at his/her agency; whether participant implemented at least one session of Connect; and whether participant implemented a complete cycle of Connect. This allowed accommodation for intraclass correlations (ICC) associated with reports from more than one facilitator at a given agency (range 0.16 – 0.54). In addition, the agency level analysis alone would not account for the fact that an agency with more facilitators has higher reliability of estimates compared to those with fewer facilitators.

## Results

### Baseline equivalence of agencies

The mean HIV/STI prevention budget for all agencies was $1,068,910; the median was $362,500 (range 0 to 35,000,000). The mean number of full time prevention staff was 18; the median was 9 (range 0 – 216). Table 1 describes organizational characteristics of participating agencies by condition. Despite variability, there were no significant differences in organizational characteristics at baseline by condition, suggesting the matched-pairs approach for randomization yielded relatively balanced groups for the trial. The actual variance accounted for due to matched pairing procedure was <0.01, which indicates that the VIFs used to estimate power were conservative statistically.

**Table 1:** **Characteristics of HIV service agencies by condition (N = 80)** ^**#**^

	**Manual-based (Traditional)**			**Web-based (Multimedia)**		
	**Mean**	**Median**	**Range**	**Mean**	**Median**	**Range**
HIV/STI prevention budget (in dollars)	593,372	280,175	0-4,000,000	1,544,449	425,000	0-35,000,000
Full-time HIV prevention staff	16	7	1-147	20	10	1-216
Part time HIV prevention staff	2	1	0-15	3	1	0-20
No. of clients receiving HIV prevention	7,207	1,192	20-81,500	3,483	1,200	50-40,000
No. of clients receiving HIV prevention	7,207	1,192	20-81,500	3,483	1,200	50-40,000
No. of clients receiving multi-session services	543	126	0-4,000	472	95	0-8,000
% of heterosexual clients	93	80	9-95	76	80	25-95
No. computers available for implementation*	8	3	1-111	8	3	0-200
No. DEBIs offered at baseline*	1	1	0-7	1	2	0-6

#rounded to whole numbers.

*individual level data from baseline assessment aggregated by agency.

### Demographic characteristics of participants

The pool of respondents included 253 agency staff (131 Web-based and 122 manual-based), with an average of three participants per agency (range 1 – 6). Most were female and over 40 years of age. The majority of participants were African American. Participants had an average of seven years of experience in HIV services, and reported high levels of confidence in using both a computer and an Internet browser (Table 2).

**Table 2:** **Sociodemographic characteristics for participants (N = 253)**

	**Manual-based (Traditional) (N = 131)**		**Web-based (Multimedia) (n = 122)**	
**Sociodemographics**	**Frequency**	**Percentage**	**Frequency**	**Percentage**
**Sex**				
Female	89	67.9	92	75.4
Male	42	32.1	30	24.6
**Age**				
18-29	18	13.7	22	18.0
30-39	35	26.7	31	25.4
40 and above	78	59.5	69	56.6
**Marital status**				
Married	45	34.4	36	29.5
Separated, Divorced, Widowed	28	21.3	28	23.0
Single	58	44.3	58	47.5
**Sexual orientation**				
Heterosexual	110	84.0	99	81.1
Gay	10	7.6	12	9.8
Lesbian	6	4.6	6	4.9
Bisexual	3	2.3	0	0
Other	1	0.8	1	.8
**Race-Ethnicity**				
African American	57	43.5	47	38.5
Hispanic	45	34.4	47	38.5
White or Caucasian	28	21.4	27	22.1
Caribbean, West Indian	9	6.9	10	8.2
Asia, SE Asia, Pac Isla.	7	5.3	3	2.5
Amer. Indian/Alaska Nat.	1	0.8	1	0.8
Other.	4	3.1	1	0.8
Born in 50 U.S. states	97	74.0	99	81.1
**Preferred language**				
English	115	87.8	106	86.9
Spanish	9	6.9	12	9.8
Other	7	5.4	4	3.2
**Education**				
High School or GED	17	13.0	23	18.9
College Technical	64	48.9	49	40.2
Graduate school	50	38.2	50	41.0
	**Mean**	**Range**	**Mean**	**Range**
Yrs in HIV/STI service	4.6	.5-13	7.1	.5-13
Yrs at agency	4.7	.5-13	2.9	.5-13
Computer confidence	8.6	2-10	9	3-10
Browser comfort	8.6	0-10	9	1-10

A x^2^ test of independence found no statistically significant (p < 0.05) association with assignment.

### Implementation over time

#### Agency level

Over 18 months, 33% of Web-based and 48% of manual-based agencies implemented at least one session. Table 3 presents the mean values for the primary outcomes by condition. As shown, for each six-month follow up period, the mean number of couples and/or sessions per agency is quite low. With the exception of ‘completed at least one cycle, trends suggest that reported implementation outcomes were higher, but slowly decreased over time among manual-based agencies, while implementation was lower but increased over time among the Web-based agencies.

**Table 3:** **Implementation over time by condition**

	**Agency level**			**Individual level**		
	**6-Month (n = 80)**	**12-Month (n = 80)**	**18-Month (n = 78)**	**6-Month (n = 228)**	**12-Month (n = 222)**	**18-Month (n = 211)**
**Number of couples per facilitator:** ***Mean (SD)***						
Manual-based	1.38 (4.79)	1.15* (2.53)	1.11 (3.34)	0.46 (2.00)	0.40** (1.11)	0.40 (1.49)
Web-based	0.53 (1.47)	0.25* (0.67)	0.98 (2.90)	0.19 (0.91)	0.09** (0.38)	0.37 (1.36)
*Overall*	0.95 (3.55)	0.70 (1.89)	1.04 (3.10)	0.33 (1.58)	0.25 (0.85)	0.38 (1.42)
**Mean of sessions per facilitator:** ***Mean (SD)***						
Manual-based	0.49 (1.27)	0.69* (1.31)	0.48 (1.21)	0.41 (1.24)	0.60* (1.52)	0.50 (1.51)
Web-based	0.23 (0.61)	0.15* (0.39)	0.37 (0.80)	0.24 (0.99)	0.19* (0.83)	0.44 (1.36)
*Overall*	0.36 (1.00)	0.42 (1.00)	0.42 (1.01)	0.33 (1.13)	0.40 (1.25)	0.47 (1.43)
**Implemented at least one session:** ***n (%)***						
Manual-based	12 (30%)	14* (35%)	10 (26%)	17 (14%)	22** (19%)	13 (12%)
Web-based	8 (20%)	6* (15%)	9 (23%)	9 (8%)	7** (7%)	12 (11%)
*Overall*	20 (25%)	20 (25%)	19 (24%)	26 (11%)	29 (13%)	25 (12%)
**Completed at least one cycle:** ***n (%)***						
Manual-based	2 (5%)	4 (10%)	6 (16%)	3 (3%)	6 (5%)	9 (8%)
Web-based	3 (8%)	1 (3%)	6 (15%)	3 (3%)	1 (1%)	8 (8%)
*Overall*	5 (6%)	5 (6%)	12 (15%)	6 (3%)	7 (3%)	17 (8%)

*p < 0.05; **p < 0.01 between conditions at each time point.

#### Participant level

Over 18 months, 17% of participants assigned to Web-based agencies and 26% of staff assigned to manual-based agencies implemented at least one session. Consistent with agency level outcomes, trends suggest that reported implementation outcomes were higher, but slowly decreased over time among manual-based agencies, while implementation was lower but increased over time among the Web-based agencies.

### Hypothesis testing: primary outcomes

#### Agency level

Table 4 demonstrates findings of multivariate analyses over time. At the agency level, only hypothesis one of four yielded significant findings.

**Table 4:** **Estimated effect of assignment to web-based versus manual-based connect at both agency and individual/participant levels: longitudinal multilevel analysis**

	**Agency level (N=80)** ^**1**^			**Individual level (N=253)** ^**2**^		
	Negative Binomial Regression Estimates			Negative Binomial Regression Estimates		
	IRR	CI	p	IRR	CI	p
***Total number of couples***						
**Average of 3 waves**	0.35*	*0.13 – 0.94*	*0.038*	0.45	0.14 – 1.45	0.181
At 6-month only	0.27*	*0.09 – 0.84*	*0.024*	0.30	0.08 – 1.10	0.070
At 12-month only	0.36*	*0.13 – 0.99*	*0.047*	0.43	0.13 – 1.40	0.163
At 18-month only	0.49	0.16 – 1.55	0.226	0.62	0.18 – 2.20	0.464
	Linear Regression Estimates			Linear Regression Estimates		
	b	CI	p	b	CI	p
***Mean number of sessions completed***						
**Average of 3 waves**	−0.31	−0.68 – 0.06	0.101	−0.25	−0.57 – 0.08	0.137
At 6-month only	−0.38	−0.80 – 0.04	0.079	−0.29	−0.66 – 0.08	0.124
At 12-month only	−0.31	−0.68 – 0.06	0.101	−0.25	−0.58 – 0.08	0.133
At 18-month only	−0.25	−0.67 – 0.18	0.257	−0.21	−0.59 – 0.17	0.279
	Logistic Regression Estimates			Logistic Regression Estimates		
	OR	CI	p	OR	CI	p
***Implement at least 1 session***						
**Average of 3 waves**	0.30	0.06 – 1.46	0.136	0.34	0.09 – 1.26	0.105
At 6-month only	0.23	0.03 – 1.50	0.124	0.23	0.05 – 1.09	0.063
At 12-month only	0.30	0.06 – 1.47	0.137	0.34	0.09 – 1.25	0.104
At 18-month only	0.40	0.06 – 2.57	0.332	0.49	0.11 – 2.22	0.354
***Completed at least 1 cycle***						
**Average of 3 waves**	0.80	0.12 – 5.55	0.819	0.71	0.13 – 3.84	0.691
At 6-month only	1.06	0.05 – 21.1	0.972	0.67	0.05 – 8.31	0.756
At 12-month only	0.82	0.09 – 7.54	0.861	0.66	0.10 – 4.44	0.667
At 18-month only	0.64	0.06 – 7.32	0.717	0.65	0.09 – 4.74	0.666

*p<0.05.

Note:

1. Measurements at the agency level:

a. *Number of couples* at the agency level = sum(# couple a facilitator reported).

b. *Mean number of sessions completed* at the agency level ***=*** sum(mean number of sessions completed by a facilitator)/(number of facilitator in an agency).

c. *Implemented at least 1 session* at the agency level = at least one facilitator reported implementing at least one session in an agency.

d. *Completed at least 1 cycle* at the agency level = at least one facilitator reported completing at least one cycle in an agency.

2. Measurements at the individual level:

a. *Number of couples* at the individual level = sum(# couple a facilitator reported).

b. *Mean number of sessions completed* at the individual level ***=*** sum(number of sessions completed with a couple)/(number of couples).

c. *Implemented at least 1 session* at the individual level = facilitators reported implementing at least one session.

d. *Completed at least 1 cycle* at the individual level = facilitators reported completing at least one cycle.

Outcome one: The outcome estimates demonstrate that, on average, the staff from Web-based agencies implemented the intervention to 35% fewer couples than those at manual-based agencies (CI, 0.13 – 0.94).

Outcomes two, three, and four: There were no differences found between conditions on the average number of sessions completed per facilitator (b = -0.31; CI, -0.068 – 0.06); the likelihood to implement at least one session (OR = 0.30; CI, 0.06 – 1.46); or the likelihood to complete at least one cycle of Connect (OR = 0.80; CI, 0.12 – 5.55).

#### Individual level

At the participant level, no significant differences were found between groups for any of the four outcomes. The outcome estimates shown in Table 4 demonstrate no differences between participants at Web-based agencies compared to those at manual-based agencies on average number of couples to whom they provided sessions (IRR = 0.45; CI, 0.14 – 1.45); number of sessions completed per facilitator (b = -0.025; CI, -0.057 – 0.08); the likelihood to implement at least one session (OR = 0.34; CI, 0.06 – 1.46); or the likelihood to complete at least one cycle of Connect (OR = 0.71; CI, 0.13 – 3.84).

### Technical assistance

Attendance for the investigator-initiated TA calls at two and four months was high (see Figure 1), however, few participating staff called the research team for TA at other times. Over the 18-month post-program period we received a total of only 28 requests, including a combination of phone calls from participants in both groups and emails from the ‘help’ button of the Web-based program.

### Barriers to implementation

The three most frequently reported barriers to implementation were economy/funding issues (47% and 53%), recruitment of couples (46% and 51%), and staff turnover (34.9% and 22%) among manual-based and Web-based conditions respectively. No significant differences between conditions were found on any reported barrier.

## Discussion

This study is the first to examine implementation of a couple-based HIV prevention intervention, and the first to test competing modalities for implementation of a behaviorally- based HIV prevention program (in this case, a CDC DEBI) among HIV services agencies. Findings did not support our hypotheses, demonstrating instead that whether measured at the level of the agency or the participants, staff trained on the Web-based program did not demonstrate significantly higher implementation compared to those trained on the manual-based approach. Agency level outcomes did demonstrate higher mean number of couples with whom staff at manual-based agencies worked compared to Web-based agencies, however, these findings were not significant at the individual level. Findings also show overall low rates of implementation, but implementation rates that are consistent with the landmark study on which the CDC DEBI approach is based [18]. Our discussion therefore focuses on potential explanations for these findings and recommendations for future research on couple-based program implementation.

As a dissemination trial, commitment from agencies to implement Connect was not an eligibility criterion. Furthermore, we did not offer additional resources or incentives for implementation. Our goal was to observe how agencies managed the process of manual or Web-based implementation with their clients post-training and TA. We did observe implementation: staff at 33% of Web-based and 48% of traditional agencies implemented sessions over 18 months; 17% of participants assigned to Web-based agencies and 26% of staff assigned to traditional agencies implemented sessions over 18 months. A smaller number implemented multiple sessions and cycles and was able to sustain use at low levels. The economic recession that began in 2008 had a devastating effect on HIV services agencies in New York State—some lost as much as 30% of their funding within three to six months. Because most participants reported their agencies were already implementing at least one DEBI, absent additional incentives or funding, more staff may have felt more comfortable implementing the traditional version of Connect, which did not require access to a computer or wireless Internet. Of note is that these low percentages are consistent with and only slightly lower than those reported in Kelly *et al*. [18,19], showing ‘adoption’ or ‘offers of service’ rates of between 36% and 60% of agencies participating in an implementation trial for another DEBI, the Peer Opinion Leader (POL) model. In this prior study the outcome was defined as ‘offers of service’ and not actual implementation, leaving us unable to examine whether actual rates of program ‘use’ are comparable.

The expected benefits of a Web-based program notwithstanding, implementation rates may reflect a time of low or dwindling resources or of considerable organizational disruption in services provision due to the recession and related cuts. Examination of data related to changes in organizational budgets over time may further inform interpretation of findings. During the study period four agencies (three manual-based and one Web-based) chose to incorporate Connect into competing grant renewals from New York State or the CDC to continue implementation. These were successful and post 18-month follow-up, these agencies continue implementation. Future trials may consider ways to offer short term resources in support of implementation or increase time spent during training sessions to how best to integrate new programs into existing service delivery or reimbursement mechanisms.

Our expectation that facilitators would use a Web-based strategy and facilitate a couple-based intervention at once may have been unrealistic and may also explain the low usage of the Web-based Connect among our agency participants. With few exceptions, the facilitators had never worked with couples. Enthusiasm during program trainings to implement a couple-based program suggested individual level commitment to implement the program. However, many expressed concerns about how they would manage couple-related interventions at their agencies. While Connect activities are simplified for easy implementation and are intended to be delivered by a wide range of facilitators, implementing a couple-based intervention presents a unique set of challenges [13,14]. Staff may have felt unduly challenged by the simultaneous demands of attending to both the new elements of a Web-based intervention and the needs of the dyad. We did not include adequate measures to examine this interpretation, so strongly recommend that future implementation trials incorporate measures sensitive to both the innovation of the program and the approach to implementation. Given the increasing attention to both couple-based and Web-based HIV prevention and the potential advantages cited [13,14] though not borne out in this study, future implementation trials might incorporate additional training and supervision for any new or novel component of implementation, particularly in the beginning stages.

Technology management issues including staff comfort and concerns about client data security and confidentiality may also account for our finding that Web-based implementation did not surpass manual-based implementation. Our choice of computer model and browser may have been unfamiliar to staff or different from the standard agency set-up to which they were accustomed. Most participating agencies employed IT staff or consultants that must approve or conduct any changes to agency-related computing; this sometimes delayed downloading and installation of required software and may have created real or imagined barriers to implementation. Further, despite almost six months of beta-testing to ensure there were no major issues with accessibility or utility of the program, ‘bugs’ in the system were reported during TA calls by some staff who tried the program early on. Together, these issues may have impeded a smooth transition to regular use of the program, or worse, led them to decide not to use the program at all. To address such issues in future trials, we recommend creating any Web-based program with support for a range of hardware and software (*i.e.*, various browsers). It would also be important to create an understanding with adopting agency staff that reporting glitches and bugs is important so that issues can be corrected quickly.

During the training for the Web-based Connect, many participants asked about the security of the program. Although we discussed the steps taken to ensure confidentiality of all program data, participating staff cited Health Information Portability and Accountability Act (HIPAA)-related concerns regarding access to the information gathered on couples engaged in the Web-based program activities. The program did not save identifiable data, rendering HIPAA concerns unwarranted. Future research should provide follow-up—with support from agency administrative staff—to clarify with staff that any programming adopted at the agency provides adequate protection of client confidentiality.

### Reported barriers to adoption across conditions

Consistent with outcomes, reported barriers to implementation did not differ by condition. The top barriers reported were lack of funding and staff turnover, consistent with existing literature on program adoption [1,20,21,41,67–70]. The next most frequently reported barrier was client recruitment, which suggests the definition of ‘couple’ may need more attention in the training sessions. During many TA calls, participants noted their agency ‘did not serve couples’ and therefore they could not offer a couple-based program. When probed, staff members reported serving individuals who were partnered. However, a shift in ideology appears to be needed in order for agency staff to see ‘couples’ as a unit of analysis that integrates with other program units ( *e.g.*, ‘individual’, ‘group’) for purposes of documentation and billing. Researchers need to work with agencies to identify how best to integrate new couple-based EBIs into new or existing services [13] and billing mechanisms.

Qualitative data collected in in-depth interviews and site visits to 10% of participating agencies should yield additional insights to further interpret these findings.

### Technical assistance

Our finding of low TA requests is not a new phenomenon. Veniegas *et al.* (2009) [21] report none of the 34 staff members interviewed among CDC-funded agencies in the Los Angeles area implementing EBIs (in a traditional format) made a single request for TA. Nevertheless, this finding raises critical questions regarding how to best meet the needs of new implementers, who would naturally have questions and need assistance, but are not yet requesting the TA available to them, and which empirical data suggest will improve implementation. What are the barriers to TA use? How do we motivate staff to request TA? Kegeles *et al.* (2012) [25], in an implementation project for MPowerment, another CDC DEBI, provided proactive TA every two weeks. After six months, requests for TA increased and then remained high for as long as two years [25]. Such a strategy may strengthen both manual- and Web-based implementation approaches and we recommend offering more targeted proactive TA during early stages of all adoption and implementation efforts.

### Limitations

Findings should be considered in light of several limitations. First, findings can only be interpreted to indicate that we found no evidence for the relationship, not evidence for lack of a relationship. The study was not powered as an equivalence trial, but rather to assess whether the Web-based approach was superior to the manual-based approach. The noted technology challenges are more anecdotal lessons learned to influence future trials, and cannot be interpreted to suggest that they were responsible for lower implementation of the Web-based program. Data were based on self-report only, which in some cases can lead to poor recall, and which can reduce confidence in findings. Operationalizing implementation is challenging; any single measure often inadequate [71]. While we expanded our definition and examination of outcomes beyond the existing model of HIV prevention implementation [18], their reliability may still be weak. A number of additional assessment questions (*e.g.* participant experience working with couples) may have strengthened analysis of findings. Finally, the loss of funds to agencies due to the recession during this trial makes it impossible to apply the findings to a more typical economic climate.

## Conclusions

Despite limitations, the study makes critical contributions to HIV prevention implementation science. Findings show the feasibility of training on and implementing both a manual-based and a Web-based version of a couple-based DEBI (or other evidence-based, manualized, behavioral intervention), and the enthusiasm of HIV services organizations and their staff to participate in implementation trials in the absence of strong economic incentives. In particular, agencies embraced the opportunity to expand service provision to include couple-based and Web-based programming. Couples-based approaches for HIV testing and counseling, as well as for more intensive primary and secondary prevention efforts are increasing. Implementation methods must be identified to best bring them to scale. Increasing interest in both of these areas of HIV prevention efficacy studies suggest the need for more careful study to inform rapid and efficient implementation of efficacious programs.

Study findings raise questions regarding the implementation of such programming in the absence of dedicated funding, however, and of barriers. Our interpretation of findings suggest that either a very poor economy, which rapidly reduced resources at participating agencies and led to low overall implementation, and (for one outcome) higher manual-based implementation; or else the combined challenge of using both a web-based curriculum and also implementing couple-based programming for the first time, may have contributed to the lack of hypothesized outcomes. Findings point to several important issues for future implementation research in HIV prevention, with couple-based programs, and/or with Web-based programming. Web-based programming should be built to support a range of browsers and beta-tested thoroughly prior to trial initiation. During trials, participants should receive more frequent proactive TA to ensure movement from the pre-implementation to implementation stages, and more time and assistance to agencies to integrate new programs into their existing program structure and reimbursement mechanisms. This should enhance support from administrators and smooth transitions for line workers managing multiple responsibilities. Support to agencies in identifying external funding for new program implementation should further reduce barriers to implementation. Future dissemination studies should also consider not only a comparison of mediums, but also the features of Web-based programming that can best support facilitators [72,73]. The task ahead is to strengthen trainings for implementation to include not just information regarding the innovation or program, but the implementation procedures and expectations (including access to resources) to best prepare agencies for the transition to couple- and Web-based approaches and ongoing support, and to identify whether, how, and when it may be efficient to use human-delivered and Web-based components of programming to achieve implementation. Future work may also consider hybrid interventions introduced in face-to-face single sessions by medical and mental health practitioners, and followed by self-administered, Web-based sessions accessed by participants or clients and their partners or other family members. Such approaches are likely given the present state of the economy, healthcare reform and changes in reimbursement for healthcare in the United States. In the end, the key factors barring most agencies post-training from successfully implementing the program remain unclear. We hope these may be borne out in ongoing analysis of the dataset, including qualitative data from agency site visits.

In the years since the initiation of this trial, the policy and best practice of HIV prevention and the use of digital technologies have changed dramatically. Interventions are being shortened to one or two sessions in the name of feasibility, economy, and practical dissemination [7]. Interventions are also now primarily targeted to individuals living with HIV and their intimate partners [6]. The Web-based program developed and implemented in this trial is the prototype for a number of HIV prevention programs currently being tested for efficacy and effectiveness [74,75]. The program’s software ‘backbone’ can be repurposed for future use in the dissemination of similar, activity-based, multi-session programs. Web-based programming will take on even more importance with the continuing economic challenges to agencies and reduced time for prevention services. Building on small successes and lessons learned here should strengthen future efforts.
